# Influence of Pyroligneous Acid on Fermentation Parameters, CO_2_ Production and Bacterial Communities of Rice Straw and Stylo Silage

**DOI:** 10.3389/fmicb.2021.701434

**Published:** 2021-07-08

**Authors:** Xiang Guo, Peng Zheng, Xuan Zou, Xiaoyang Chen, Qing Zhang

**Affiliations:** ^1^Guangdong Key Laboratory for Innovative Development and Utilization of Forest Plant Germplasm, State Key Laboratory for Conservation and Utilization of Subtropical Agro-Bioresources, Guangdong Province Research Center of Woody Forage Engineering Technology, Guangdong Research and Development Center of Modern Agriculture (Woody forage) Industrial Technology, South China Agricultural University, Guangzhou, China; ^2^College of Horticulture, South China Agricultural University, Guangzhou, China

**Keywords:** greenhouse gas, bacterial community, rice straw, stylo, fermentation quality

## Abstract

Carbon dioxide (CO_2_) is a primary greenhouse gas and the main cause of global warming. Respiration from plant cells and microorganisms enables CO_2_ to be produced during ensiling, a method of moist forage preservation applied worldwide. However, limited information is available regarding CO_2_ emissions and mitigation during ensiling. Pyroligneous acid, a by-product of plant biomass pyrolysis, has a strong antibacterial capacity. To investigate CO_2_ production and the influence of pyroligneous acid, fresh stylo, and rice straw were ensiled with or without 1% or 2% pyroligneous acid. Dynamics of the fermentation characteristics, CO_2_ production, and bacterial communities during ensiling were analyzed. Pyroligneous acid increased the lactic acid content and decreased the weight losses, pH, ammonia-N content, butyric acid content, and coliform bacterial numbers (all *P* < 0.05). It also increased the relative abundance of *Lactobacillus* and decreased the relative abundances of harmful bacteria such as *Enterobacter* and *Lachnoclostridium*. Adding pyrolytic acids reduced the gas production, especially of CO_2_. It also increased the relative abundances of CO_2_-producing bacterial genera and of genera with the potential for CO_2_ fixation. In conclusion, adding pyroligneous acid improved the fermentation quality of the two silages. During ensiling, CO_2_ production was correlated with bacterial community alterations. Using pyroligneous acid altered the bacterial community to reduce CO_2_ production during ensiling. Given the large production and demand for silage worldwide, application of pyroligneous acid may be an effective method of mitigating global warming via CO_2_ emissions.

## Introduction

Carbon dioxide (CO_2_), a primary greenhouse gas, has received increasing attention in the past two decades and has become a priority because of its low-carbon and sustainable development worldwide (Ray et al., 2019). Approximately, 15% of anthropogenic greenhouse gas emissions are generated by animal husbandry production (Adegbeye et al., 2019). In recent years, considerable efforts have been made to reduce greenhouse gas emissions from animal husbandry production and manure treatment. Ensiling is a traditional method of conserving forage, and silage is used as an important nutrient feed source for ruminants worldwide (Dunière et al., 2013). In China, silage production is reported to exceed 280 million tons annually (Liu Z. et al., 2020) and is expected to further increase as the consumption of livestock products, such as milk and beef, increases. Metabolism of microorganisms and plant cells in silage leads to gas emissions, of which, CO_2_ is the main gas produced. Cai et al. (1997) reported that after 60 days of fermentation, gas production exceeds 6.0 L/kg of fresh matter. However, McEniry et al. (2011) reported that CO_2_ production mainly occurs in the early stages of ensiling, which constitutes > 60% of all the gas produced. CO_2_ production during ensiling leads to nutrient loss from the silage and impacts the greenhouse effect, which affects the earth’s ecology. However, little research has been conducted on CO_2_ emissions from silage.

Pyroligneous acid, a by-product of plant biomass pyrolysis, is a complex, condensed, crude, and highly oxygenated aqueous liquid fraction generated during wood charcoal production (Li et al., 2018). Pyroligneous acid consists of more than 200 compounds, including furan, organic acids, esters, phenols, alcohols, and pyran derivatives (Liu X. et al., 2020), and is recycled in many areas as an important commercially valuable resource. Pyroligneous acid is beneficial to agriculture and has been used as an insecticide, fertilizer, soil enhancer, animal feed supplement, and source of smoke flavoring for food (Zheng et al., 2020). Pyroligneous acid has strong antibacterial abilities owing to the presence of organic acids and phenolic compounds. Previous studies have shown that pyroligneous acid prevents the activities of microorganisms such as *Pseudomonas*, *Escherichia*, *Staphylococcus*, *Aspergillus*, and *Candida*, which are abundant during ensiling (de Souza Araújo et al., 2018; Suresh et al., 2019; Bai et al., 2020; Wu et al., 2020). However, the effects of pyroligneous acid on bacterial communities during ensiling remain unknown.

We hypothesized that adding pyroligneous acid during ensiling would reduce CO_2_ production by altering the bacterial communities. We analyzed the fermentation quality, CO_2_ production, and bacterial communities of fresh stylo and rice straw ensiled with pyroligneous acid.

## Materials and Methods

### Silage Preparation

Stylo (*Stylosanthes guianensis*, CIAT 184) and rice (*Oryza sativa* L., Huahang 38) were planted without herbicide or fertilizer application in an experimental field of South China Agricultural University (23.24°N, 113.64°E, Guangzhou, China). Stylo (at the bloom stage) and rice straw (at the seed-harvesting stage) were harvested on August 08, 2020 and August 18, 2020, respectively. The two fresh materials were mixed and chopped to 1–2 cm by hand with a paper cutter, then treated with 1% or 2% pyroligneous acid based on fresh matter. Pyroligneous acid was obtained from blended wood waste and filtered through a 0.45-μm cellulose acetate membrane, similar to that reported by Zhang Y. et al. (2020). Approximately, 100 g of silage materials were packed and compressed manually into plastic-film bags (12 bags per treatment), sealed with a vacuum sealer, and stored indoors at 27–32°C. Silage samples from three bags were randomly collected after 3, 7, 14, and 30 days of fermentation, and the fermentation, gas production, and bacterial community parameters were determined.

### Determination of Gas Production and CO_2_ Concentration

The silage bag volume was measured in a 5000-mL beaker in a constant 25°C water bath. Gas production was then calculated using the difference in the volumes before and after silage (Cai et al., 1997). One microliter of the gas sample was collected with a microsyringe and injected into a gas chromatograph (Shimadzu GC-20A) to determine the CO_2_ concentration. The CO_2_ was separated on a molecular Sieve 5A and Porapak N column, with an oven temperature of 60°C for 5.5 min. The temperatures of the injector and detector were held at 100°C and 170°C, respectively. The quantitation limit of CO_2_ in the gas chromatograph is 0.1% (v/v) using this method.

### Fermentation Characteristics Analysis

The methods used to analyze the fermentation characteristics were similar to those used in our previous studies (Wang et al., 2018; He et al., 2020). Briefly, 20 g (including raw material and silage) of stylo and rice straw were taken randomly, soaked in 180 mL of sterile 0.9% saline for ∼15 min, and serially diluted from 10^–1^ to 10^–6^ on a clean bench. Lactic acid bacteria (LAB) and coliform bacteria were cultured and estimated using deMan-Rogosa-Sharpe agar and violet red bile agar at 30°C for 2 days. Yeast and mold were cultured and determined on Rose-Bengal agar for 2 days at 28°C. The 20 g of each silage sample were homogenized with 180 mL of distilled water for 18 h at 4°C, then filtered through four layers of cheesecloth and filter paper. A glass-electrode pH meter was used to immediately measure the pH of this filtrate. Organic acid contents were determined using high-performance liquid chromatography as described by Wang et al. (2018). The dry matter content was measured immediately after drying the samples at 65°C using an electric dryer equipped with an air blower. The ammonia-N content was determined using a phenol-hypochlorite assay.

### Bacterial Community Sequencing Analysis

The total bacterial DNA was extracted from the silage samples using a DNA kit (Omega Biotek, Norcross, GA, United States) following the manufacturer’s instructions and using specific steps as reported by Bai et al. (2020). The V3-V4 regions of the 16S rDNA were amplified using the primers, 341F: CCTACGGGNGGCWGCAG and 806R: GGACTACHVGGGTATCTAAT, and PCR was conducted using a 50-μL reaction mixture consisting of 1.5 μL of 5 μM of each primer, 1 μL KOD polymerase, 5 μL 10 × KOD buffer, 100 ng template DNA, and 5 μL of 2.5 mM dNTPs per the procedures of He et al. (2020). After purification and quantification, an Illumina HiSeq 2500 Sequencing System (Illumina, Inc., San Diego, CA, United States) was used for the PCR sequencing, and the raw sequences were analyzed as described by Wang et al. (2018). The bioinformatic data were examined via the free online platform at http://www.omicshare.com/tools by GENE *DENOVO*, and the QIIME bioinformatic pipeline^1^ and principal coordinate analysis (PCoA) were used to calculate the α-diversity and β-diversity, respectively. The relative abundances of different bacterial communities at the phylum and genus levels were analyzed. The sequencing data were deposited in the Sequence Read Archive (SRA) under the accession number PRJNA735102.

### Statistical Analysis

Statistical analysis was performed using SPSS 20.0 software, and the threshold for statistical significance was *P* < 0.05. All microbial count data were log_10_-transformed, and all figures were constructed using Adobe Illustrator CS 6.0.

## Results

### Fermentation Properties of Stylo and Rice Straw Silage During Ensiling

Tables 1, 2 show the fermentation parameter dynamics of the stylo and rice straw silage. The lactic acid, acetic acid, and propionic acid contents increased, and the weight loss; pH; numbers of coliform bacteria, yeast, and mold and ammonia-N content decreased during ensiling (all *P* < 0.05).

**TABLE 1:** Fermentation characteristics of stylo ensiled with or without pyroligneous acid (PA).

		**Ensiling time (d)**					**SEM**	**Significance**		
		**3**	**7**	**14**	**30**	**Mean**		**T**	**A**	**T×A**
Weight loss (%)	CK	0.39^a^	0.83^a^	1.40^a^	2.71^a^	1.33^a^	0.761	<0.01	<0.01	<0.01
	1% PA	0.18^b^	0.40^b^	0.79^b^	1.88^b^	0.81^b^				
	2% PA	0.15^b^	0.34^b^	0.62^b^	1.32^C^	0.68^C^				
	Mean	0.25^D^	0.54^C^	0.93^b^	1.97^a^					
DM (%)	CK	30.3	27.9^b^	28.0^b^	27.0^b^	28.3^b^	1.15	<0.01	<0.01	0.06
	1% PA	30.6	28.9^a^	29.5^a^	29.3^a^	29.3^a^				
	2% PA	30.4	29.1^a^	28.6^AB^	28.9^a^	29.6^a^				
	Mean	30.4^a^	28.6^b^	28.7^b^	28.4^b^					
pH	CK	6.43^a^	5.70^a^	5.69^a^	5.49^a^	5.83^a^	0.520	<0.01	<0.01	0.026
	1% PA	5.77^b^	5.04^b^	4.75^b^	4.89^b^	5.11^b^				
	2% PA	5.43^C^	4.97^b^	4.84^b^	4.71^b^	4.99^C^				
	Mean	5.87^a^	5.24^b^	5.10^C^	5.03^C^					
Lactic acid (% DM)	CK	0.93	0.80^b^	0.65^C^	0.71^b^	0.77^C^	0.16	0.012	<0.01	<0.01
	1% PA	1.00	1.05^a^	0.96^b^	0.82^b^	0.96^b^				
	2% PA	1.05	1.02^a^	1.10^a^	1.08^a^	1.06^a^				
	Mean	0.99^a^	0.96^AB^	0.90^bC^	0.87^C^					
Acetic acid (% DM)	CK	0.44^b^	0.69^b^	0.77	0.95	0.71^b^	0.208	0.067	<0.01	<0.01
	1% PA	1.05^a^	1.08^a^	0.92	0.74	0.95^a^				
	2% PA	1.13^a^	1.07^a^	0.89	0.82	0.98^a^				
	Mean	0.87^AB^	0.95^a^	0.86^AB^	0.84^b^					
Propionic acid (% DM)	CK	ND	ND	ND	ND	ND	–	–	–	–
	1% PA	ND	ND	ND	ND	ND				
	2% PA	ND	ND	ND	ND	ND				
	Mean	ND	ND	ND	ND					
Butyric acid (% DM)	CK	ND	0.41	0.49	1.16	0.68	0.392	<0.01	–	–
	1% PA	ND	ND	ND	ND	ND				
	2% PA	ND	ND	ND	ND	ND				
	Mean	ND	0.41^b^	0.49^b^	1.16^a^					
Lactic acid bacteria (Log_10_ cfu/g FM)	CK	7.90^a^	7.81	7.48^b^	7.20^b^	7.60^a^	0.696	<0.01	<0.01	<0.01
	1% PA	6.80^b^	7.65	8.11^a^	7.29^b^	7.46^a^				
	2% PA	5.55^C^	7.56	7.87^AB^	7.98^a^	7.24^b^				
	Mean	6.75^C^	7.67^a^	7.82^a^	7.49^b^					
Coliform bacteria (Log_10_ cfu/g FM)	CK	7.79^a^	7.52^a^	6.94^a^	<3.00	7.42^a^	1.624	0.022	<0.01	0.443
	1% PA	5.43^b^	4.90^b^	3.50^b^	<3.00	4.61^b^				
	2% PA	<3.00	<3.00	<3.00	<3.00	<3.00				
	Mean	6.85^a^	6.21^AB^	5.56^b^	<3.00					
Yeasts and moulds (Log_10_ cfu/g FM)	CK	<2.00	<2.00	<2.00	<2.00	<2.00	–	–	–	–
	1%PA	<2.00	<2.00	<2.00	<2.00	<2.00				
	2% PA	<2.00	<2.00	<2.00	<2.00	<2.00				
	Mean	<2.00	<2.00	<2.00	<2.00					
Ammonia-N (% TN)	CK	6.59^a^	8.89^a^	11.4^a^	14.3^a^	10.3^a^	4.33	<0.01	<0.01	<0.01
	1% PA	1.33^b^	1.89^b^	3.43^b^	4.89^b^	2.89^b^				
	2% PA	1.01^b^	1.25^C^	1.82^C^	3.00^b^	1.77^C^				
	Mean	2.98^D^	4.01^C^	5.53^b^	7.41^a^					

*DM, dry matter; FM, fresh matter; TN, total N; SEM, standard error of means; ND, not detected; T, time of ensiling; A, additives; T×A, interaction of ensiling time and additives. Means with different letters in the same column (a–d) or row (A–D) indicate a significant difference (*P* <0.05).*

**TABLE 2:** Fermentation characteristics of rice straw ensiled with or without pyroligneous acid (PA).

		**Ensiling time (d)**					**SEM**	**Significance**		
		**3**	**7**	**14**	**30**	**Mean**		**T**	**A**	**T×A**
Weight loss (%)	CK	0.51^a^	1.08^a^	1.67^a^	2.75^a^	1.50^a^	0.844	<0.01	<0.01	0.374
	1% PA	0.29^b^	0.79^b^	1.30^b^	2.44^b^	1.20^b^				
	2% PA	0.21^b^	0.61^C^	1.29^b^	2.43^b^	1.22^b^				
	Mean	0.35^D^	0.83^C^	1.42^b^	2.54^a^					
DM (%)	CK	39.6	37.3	38.3	37.3^b^	38.1	1.11	0.055	0.175	0.059
	1%PA	38.6	38.6	38.5	39.6^a^	38.8				
	2% PA	39.0	37.8	38.9	37.4^b^	38.3				
	Mean	39.1^a^	37.9^b^	38.6^AB^	38.1^b^					
pH	CK	5.28^a^	524^a^	5.22^a^	4.82^a^	5.14^a^	0.217	<0.01	<0.01	<0.01
	1% PA	4.85^b^	4.82^b^	4.82^b^	4.75^AB^	4.81^b^				
	2% PA	4.96^b^	4.76^C^	4.72^b^	4.69^b^	4.78^b^				
	Mean	5.03^a^	4.94^b^	4.92^b^	4.75^C^					
Lactic acid (% DM)	CK	0.60^b^	0.65^b^	0.61^b^	0.68	0.63^b^	0.058	<0.01	<0.01	0.089
	1% PA	0.67^a^	0.73^a^	0.74^a^	0.73	0.71^a^				
	2% PA	0.64^AB^	0.75^a^	0.72^a^	0.74	0.72^a^				
	Mean	0.63^C^	0.71^AB^	0.69^b^	0.72^a^					
Acetic acid (% DM)	CK	0.28^b^	0.44^C^	0.58	0.81^a^	0.53^b^	0.126	<0.01	0.034	<0.01
	1% PA	0.47^a^	0.49^b^	0.55	0.59^b^	0.52^b^				
	2% PA	0.48^a^	0.61^a^	0.58	0.59^b^	0.57^a^				
	Mean	0.41^D^	0.51^C^	0.57^b^	0.66^a^					
Propionic acid (% DM)	CK	ND	0.18	0.34^a^	0.52^a^	0.35^a^	0.150			
	1%PA	ND	ND	ND	0.06^b^	0.06^b^				
	2% PA	ND	ND	ND	0.03^b^	0.03^b^				
	Mean	ND	0.18^b^	0.34^a^	0.23^b^					
Butyric acid (% DM)	CK	ND	ND	ND	0.01	0.01	0.002	<0.01	-	-
	1%PA	ND	ND	ND	ND	ND				
	2% PA	ND	ND	ND	ND	ND				
	Mean	ND	ND	ND	0.01					
Lactic acid bacteria (Log_10_ cfu/g FM)	CK	8.81	8.69	8.20	7.77	8.36	0.488	<0.01	0.193	0.947
	1%PA	8.85	8.70	8.20	7.94	8.42				
	2% PA	8.63	8.58	8.14	7.50	8.21				
	Mean	8.76^a^	8.65^a^	8.18^b^	7.74^C^					
Coliform bacteria (Log_10_ cfu/g FM)	CK	7.61^a^	7.60^a^	5.93^a^	<3.00	7.05^a^	0.818	<0.01	<0.01	0.314
	1% PA	6.72^b^	6.61^AB^	5.57^b^	<3.00	6.30^b^				
	2% PA	6.37^b^	5.79^b^	<3.00	<3.00	6.08^b^				
	Mean	6.90^a^	6.67^a^	5.75^b^	<3.00					
Yeasts (Log_10_ cfu/g FM)	CK	4.76	4.43	3.14	<2.00	4.11	0.690	<0.01	0.885	0.173
	1%PA	4.35	4.24	3.86	<2.00	4.05				
	2% PA	4.62	3.95	3.14	<2.00	4.00				
	Mean	4.58^a^	4.21^a^	3.38^b^	<2.00					
Moulds (Log_10_ cfu/g FM)	CK	4.20	<2.00	<2.00	<2.00	4.20	0.373	-	-	-
	1%PA	<2.00	<2.00	<2.00	<2.00	<2.00				
	2% PA	<2.00	<2.00	<2.00	<2.00	<2.00				
	Mean	4.20	<2.00	<2.00	<2.00					
Ammonia-N (% TN)	CK	4.31^a^	10.4^a^	14.7^a^	17.6^a^	11.7^a^	4.700	<0.01	<0.01	<0.01
	1% PA	3.42^b^	5.26^b^	7.62^b^	8.59^b^	6.22^b^				
	2% PA	1.97^C^	3.38^C^	4.74^C^	6.41^C^	4.12^C^				
	Mean	3.23^D^	6.35^C^	9.02^b^	10.9^a^					

*DM, dry matter; FM, fresh matter; TN, total N; SEM, standard error of means; ND, not detected; T, time of ensiling; A, additives; T×A, interaction of ensiling time and additives. Means with different letters in the same column (a–d) or row (A–D) indicate a significant difference (*P* <0.05).*

### Bacterial Community Dynamics During Ensiling

**FIGURE 1:**
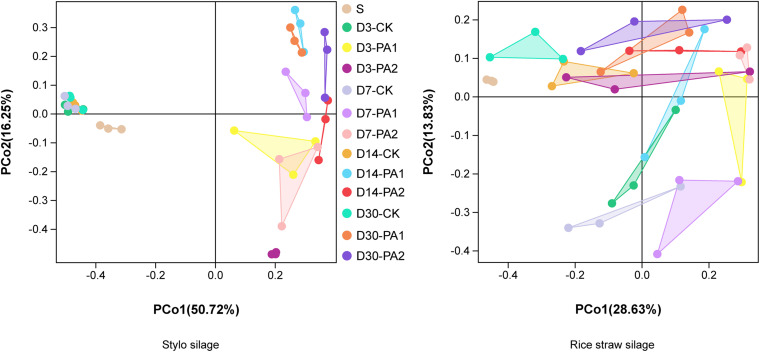
Principal coordinate analysis of bacterial communities in stylo and rice straw before and after ensiling for 3, 7, 14, and 30 days with or without 1% or 2% pyroligneous acid.

**FIGURE 2:**
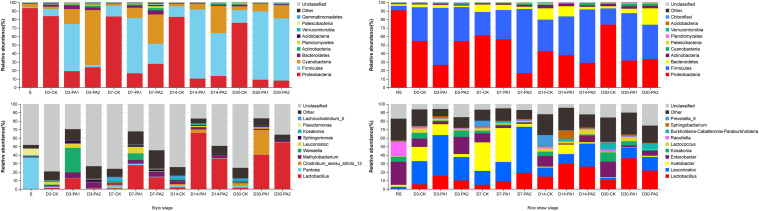
Relative abundances of bacterial communities at the phylum and genus levels in stylo and rice straw before and after ensiling for 3, 7, 14, and 30 days with or without 1% or 2% pyroligneous acid.

**FIGURE 3:**
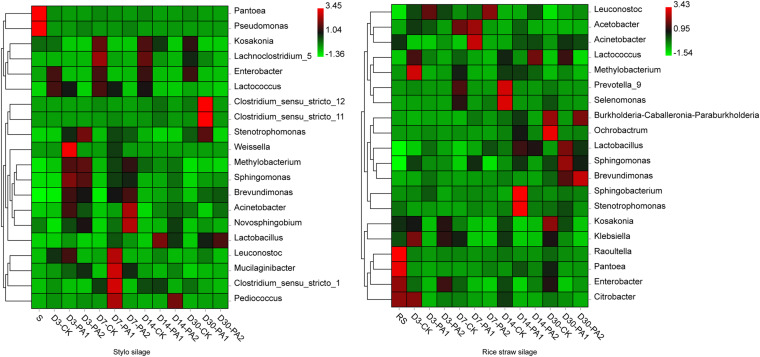
Heatmap of bacterial communities at the genus level in stylo and rice straw before and after ensiling for 3, 7, 14, and 30 days with or without 1% or 2% pyroligneous acid.

**FIGURE 4:**
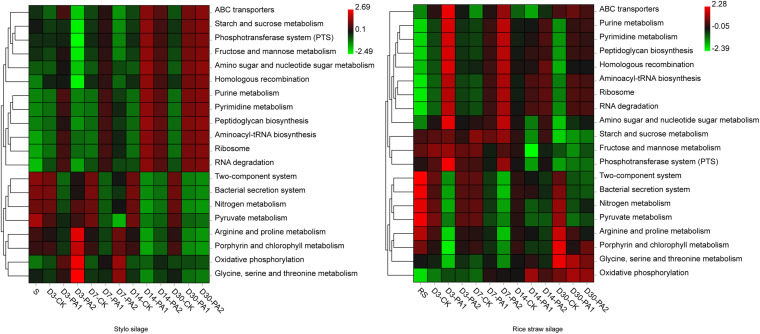
Heatmap of 16S rRNA gene-predicted functional profiles of the microbial communities in stylo and rice straw before and after ensiling for 3, 7, 14, and 30 days with or without 1% or 2% pyroligneous acid.

**FIGURE 5:**
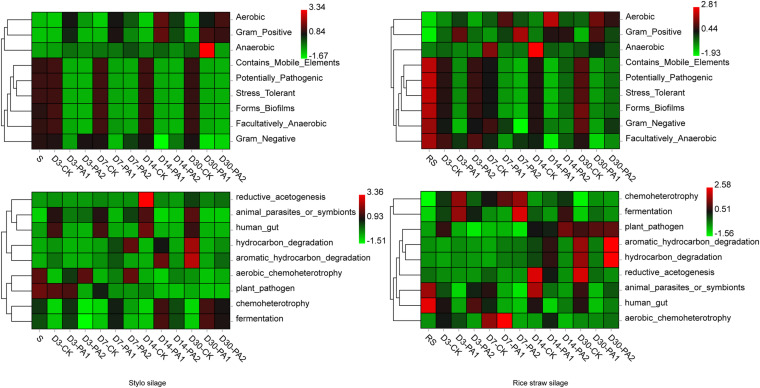
Heatmap of phenotypic characteristics and predicted functional profiles of detected bacterial communities in stylo and rice straw before and after ensiling for 3, 7, 14, and 30 days with or without 1% or 2% pyroligneous acid.

In the stylo silage treatments, PCoA1 and PCoA2 accounted for 28.6% and 50.7% of the total variance; in the rice straw silage treatments, PCoA1 and PCoA2 accounted for 13.8% and 16.3% of the total variance, respectively (Figure 1). Figure 2 shows the relative bacterial community abundances at the phylum and genus levels. Proteobacteria and Firmicutes were the dominant phyla in both the rice straw and stylo silage, and their relative abundances decreased during ensiling. The abundance of *Lactobacillus* in the silage after pyroligneous acid treatment was higher than that in the control silage (Figures 2, 3). Adding 1% pyroligneous acid increased the relative abundances of *Leuconostoc* on days 3 and 7 in the stylo silage, and adding 2% pyroligneous acid increased the relative abundances of *Leuconostoc* and *Lactococcus* in the rice straw silage on days 7 and 14, respectively (Figures 2, 3). In the stylo silage, the relative abundance of *Novosphingobium* spp. increased, and the relative abundances of *Enterobacter* and *Kosakonia* decreased after pyroligneous acid treatment (Figures 2, 3). Serine, arginine, nitrogen, glycine, proline, and threonine metabolism decreased after adding pyroligneous acid (Figure 4). Thus, adding pyroligneous acid may reduce potential pathogens on forage surfaces during ensiling (Figure 5). Adding pyroligneous acid also enhanced fermentation and reduced hydrocarbon degradation.

### Gas and CO_2_ Production During Ensiling

**FIGURE 6:**
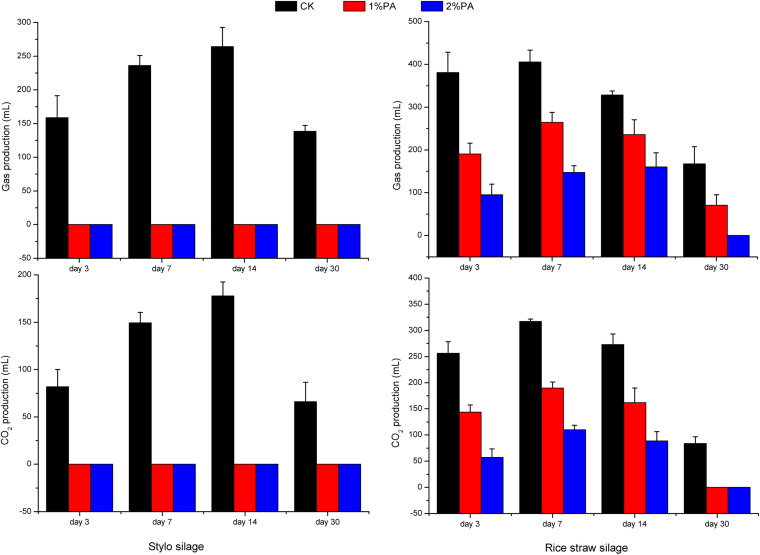
Gas production and CO_2_ production of stylo and rice straw silage after fermentation for 3, 7, 14, and 30 days with or without 1% or 2% pyroligneous acid.

Gas was produced in accordance with CO_2_ production and was drastically increased in both silages during week 1 (Figure 6). CO_2_ production reached its maximum on day 14 (178 mL) in the naturally fermented stylo and on day 7 (317 mL) in the rice straw silage without additives owing to the different plant species and microorganisms in the two materials. Adding pyroligneous acid decreased the gas and CO_2_ production in both silages. Adding pyroligneous acid to the laboratory silos reduced the CO_2_ content of 100 g of stylo silage by 66 mL and of 100 g of rice straw silage by 84 mL.

The gas and CO_2_ production increased substantially in the early stages of ensiling. Pyroligneous acid treatment increased the relative abundances of *Leuconostoc* spp. and decreased the pH and relative abundances of *Lactococcus* spp. After 14 days of ensiling without adding pyroligneous acid, the relative abundances of *Lactococcus* spp. decreased in the stylo silage. Adding 1% pyroligneous acid to the silage on days 14 and 30 increased and enhanced the relative abundances of *Clostridium* spp. (Figures 2, 3). *Lachnoclostridium* was active on day 7 but was weakened during ensiling. The relative abundance of *Lachnoclostridium* also decreased after pyroligneous acid treatment. The relative abundance of *Prevotella* in the rice straw silage was higher on days 7 and 14 than on days 3 and 30 (Figure 3). Pyroligneous acid treatment decreased the relative abundances of *Selenomonas*, *Enterobacter*, *Prevotella*, and *Citrobacter*. Adding pyroligneous acid to the stylo silage increased the relative abundance of *Methylobacterium*.

## Discussion

### Fermentation Properties of Stylo and Rice Straw Silage During Ensiling

Silage, a traditional method of preserving fresh forage, is very common and is used in ruminant production worldwide (Araújo et al., 2020). During ensiling, epiphytic microorganisms (mostly LAB) start fermentation under anaerobic conditions and produce lactic acid, causing the pH to decrease, inhibiting harmful microorganisms, and ultimately preserving the moist forage (Weinberg and Muck, 1996). Forages such as stylo and rice straw are difficult to directly ensile owing to their low water-soluble carbohydrate content and high abundance of undesirable microorganisms (Wang C. et al., 2019; He et al., 2020). Without pyroligneous acid, the fermentation quality of the stylo and rice straw silage was low because of the relatively high pH. Organic acids, especially acetic acid, are the main components of pyroligneous acid (Zhang Y. et al., 2020), which may partly explain the lower pH (*P* < 0.05) and higher acetic acid content (*P* < 0.05) in the present study in the pyroligneous acid-treated silages compared with those in the control silage. Plant cell respiration and microorganismal activities lead to nutrient losses during ensiling, especially in the early stages, and pyroligneous acid can decrease these losses possibly by direct acidification, which inhibits plant cell respiration and microbial activities. Acetic acid can be used to improve the aerobic stability of silage (Zhang et al., 2018). Therefore, the addition of pyroligneous acid might be helpful to improves the aerobic stability of silage and further study is needed.

Wang Y. et al. (2019) reported that lactic acid produced by LAB fermentation decreased the pH in the early stage of ensiling. In the current study, the lactic acid content increased in pyroligneous acid-treated silage. Butyric acid is an undesirable product in silage owing to the nutrient loss resulting from secondary fermentation caused by clostridial activity (McDonald et al., 1991). Pyroligneous acid treatment significantly decreased the butyric acid content (*P* < 0.05), possibly by inhibiting the activities of *Clostridium* spp. owing to the reduced pH after adding pyroligneous acid. Protein degradation results in non-protein-N and ammonia-N accumulation in silage, which have low utilization efficiency in ruminants, thus declining the silage quality (He et al., 2019).

Animal excretion negatively impacts the economy and ecology. Therefore, effective measures should be taken to reduce or prevent proteolysis in silage. The ammonia-N content is an important index of protein decomposition during ensiling (Pahlow et al., 2003) and is influenced by coliform bacterial activity. In this study, adding pyroligneous acid decreased the ammonia-N content (*P* < 0.01), which was consistent with the decreased coliform bacterial numbers. The mold numbers in the rice straw silage also decreased with pyroligneous acid treatment, similar to that reported by Jung (2007) and Suresh et al. (2019), who found that pyroligneous acid exerted growth-inhibiting effects on fungi such as *Aspergillus* spp.

### Bacterial Community Dynamics During Ensiling

In this study, the improved fermentation quality was in accordance with the changes in the bacterial community during ensiling. The unweighted PCoA findings reflected the distinctions in the bacterial communities among treatments. Silages ensiled without additives were separated from the additive-treated silages, suggesting that pyroligneous acid affected the bacterial communities in the silage. *Lactobacillus* is the major LAB in silage and can grow rapidly and produce lactic acid using water-soluble carbohydrates as substrates. *Lactobacillus* can also decrease the pH after oxygen is exhausted by plant cells and aerobic microorganisms in the early stage of ensiling. *Leuconostoc* and *Lactococcus* are main lactate-producing bacteria during ensiling and are usually used for fermentation in the early stage to effectively improve fermentation quality (Pahlow et al., 2003; Ni et al., 2018). *Novosphingobium*, a Gram-negative chemo-organotrophic bacterium, degrades various aromatic hydrocarbons. The increase in *Novosphingobium* spp. in the present study in the pyroligneous acid-treated stylo silage might have been due to the high aromatic hydrocarbon content in the pyroligneous acid (Zheng et al., 2020).

*Enterobacter* competes with LAB for oxygen and fermentation substrates and is an undesirable microorganism during ensiling; *Enterobacter* slows the decrease in the pH and increases protein degradation (Wang Y. et al., 2019). *Kosakonia* has characteristics similar to those of *Enterobacter* (Li et al., 2016), and its abundance in stylo silage was also reduced by pyroligneous acid treatment, possibly owing to the rapid decline in the pH that was inhibiting its growth. Notably, undesirable microorganisms such as *Enterobacter* can produce ammonia-N by fermenting amino acids. Dunière et al. (2013) reported that some amino acid decarboxylation may lead to accumulation of biogenic amines, which negatively affects animal health. Ensiling and adding pyroligneous acid improve fermentation quality owing to compounds such as organic acids, carbonyls, and phenolic derivatives—as well as the strong antimicrobial and antiviral activities of pyroligneous acid (Li et al., 2018; Suresh et al., 2019). Pyroligneous acid is reported to inhibit undesirable microorganisms, including *Escherichia*, *Enterobacter*, *Pseudomonas*, and *Listeria*, similar to the findings of the present study. Antibiotics are responsible for the present spread of multi-antibiotic-resistant bacteria, and many countries such as China ban the use of antibiotics in animal feed. Thus, as a natural antibacterial agent, pyroligneous acid may be a good alternative to conventional drugs in livestock farming.

### Gas and CO_2_ Production During Ensiling

In the early stage of silage fermentation, CO_2_ and other gases are produced via respiration by plant cells and microorganisms, leading to gas accumulation. However, the gases produced from ensiling have attracted little attention although they may cause nutrient losses and the add to the greenhouse effect. Gas and CO_2_ production drastically increased in both silages in week 1, which is similar to the findings of McEniry et al. (2011). *Lactobacillus casei* and *L. plantarum* in sorghum silage can reduce gas production; thus, rapid acidification during ensiling may decrease plant cell respiration and bacterial community changes (Cai et al., 1997). Gas production by both silages increased or decreased slowly after 7 days, possibly due to the environmental hypoxia and acidification inhibiting plant cell respiration and gas-producing bacteria. Silages are fermented for approximately, 30 days before being opened and fed to ruminants. In this study, adding pyroligneous acid reduced the CO_2_ content and thus might be an effective method of reducing greenhouse gas emissions and mitigating climate change.

Gas and carbon dioxide production increased in the early stage of ensiling, which might be correlated with the relatively high abundances of *Leuconostoc* (3.41% and 27.2% in the stylo and rice straw silage on day 3, respectively). Adding pyroligneous acid increased the relative abundances of *Leuconostoc* spp., which was inconsistent with the reduction in CO_2_ production in the pyroligneous acid-treated silages. In the present study, the pH and relative abundances of *Lactococcus* spp. decreased with pyroligneous acid treatment, possibly owing to the decreased CO_2_ production in the pyroligneous acid-treated silages. Bacterial community alterations may explain the changes and reduction in CO_2_ production during fermentation after adding pyroligneous acid. Zhai and Pérez-Díaz (2020) reported that *Leuconostocaceae* are the most important microorganisms that produce CO_2_ during anaerobic fermentation. The relative abundances of *Leuconostoc* spp. increased, and CO_2_ production decreased in pyroligneous acid-treated silages possibly because CO_2_ production during fermentation is a complex process, and many other bacteria are involved. One study reported that increasing the initial pH from 6.0 to 6.8 significantly increased the CO_2_ production rate of *Lactococcus* spp. (Andersen et al., 2005).

*Clostridium* spp. are considered a major CO_2_ source in silage (Pahlow et al., 2003). The relative abundances of *Clostridium* spp. increased, possibly because some *Clostridium* spp. are autotrophic acetogenic bacteria that can produce important chemicals and fuels by using CO_2_ (Zhang L. et al., 2020). *Lachnoclostridium*, a newly defined genus under the highly polyphyletic class *Clostridia*, showed a decreased relative abundance after pyroligneous acid treatment, consistent with that in CO_2_ production. Furthermore, *Selenomonas*, *Enterobacter*, *Prevotella*, and *Citrobacter* may also be sources of CO_2_ production during ensiling. Chen and Wolin (1977) presumed that *Selenomonas* spp. could ferment carbohydrates mainly to organic acids and CO_2_; Converti and Perego (2002) reported that CO_2_ is a major product of *Enterobacter aerogenes*; Emerson and Weimer (2017) reported that some *Prevotella* strains could produce CO_2_ as the main product, and Lee et al. (2018) found that *Citrobacter amalonaticus* could produce CO_2_ and H_2_. In recent years, various approaches have been developed to reduce CO_2_ emissions, among which, exploration of bacterial strains with CO_2_ sequestration capacity might be effective. von Borzyskowski et al. (2018) reported that *Methylobacterium* could generate biomass from CO_2_ using a heterologous Calvin-Benson-Bassham cycle. Okyay and Rodrigues (2015) considered that *Brevundimonas*, *Sphingobacterium*, *Pseudomonas*, and *Acinetobacter* strains can sequester CO_2_. Furthermore, the abundance of *Stenotrophomonas*, which can fix CO_2_, has been reported to increase in pyroligneous acid-treated silages (Okyay et al., 2016). Wang et al. (2020) added biochar and slag to paddy fields and observed a higher relative abundance of *Sphingomonas* and lower CO_2_ emissions than those of the control fields and speculated that that *Sphingomonas* could reduce CO_2_ emissions and sequestrate soil C. The increases in the relative abundances of *Methylobacterium*, *Brevundimonas*, *Sphingobacterium*, *Pseudomonas*, *Stenotrophomonas*, and *Acinetobacter* in pyroligneous acid-treated silage might also explain the reduced CO_2_ production. Therefore, increasing the abundances of microorganisms that can sequester CO_2_ may reduce greenhouse gas emissions and nutrient loss, and it might be possible to isolate such microorganisms from silage. Notably, CO_2_ is used in many food products because high levels can inhibit the growth of some microorganisms. For example, CO_2_ treatment was reported to reduce the abundances of detrimental bacteria, such as *Pseudomonas* and *Serratia*, in milk (Lo et al., 2016). Similarly, in this study, higher abundances of *Pseudomonas* were observed in silage without pyroligneous acid treatment. Thus, adding pyroligneous acid may reduce CO_2_ production by changing the bacterial communities in rice straw and stylo silage.

## Conclusion

Pyroligneous acid improved the fermentation quality of rice straw and stylo silage by increasing the lactic acid content and decreasing the weight losses, ammonia-N content, pH, butyric acid content, and coliform bacterial numbers. Additionally, pyroligneous acid increased the relative abundance of *Lactobacillus* and decreased that of undesirable bacteria such as *Enterobacter* and *Lachnoclostridium*. CO_2_ production was reduced during ensiling, and pyroligneous acid treatment increased the relative abundances of CO_2_-fixing genera. Given the immense production and demand for silage worldwide, application of pyroligneous acid may be an effective means of alleviating climate change caused by CO_2_ emissions.

## Data Availability Statement

The datasets presented in this study can be found in online repositories. The names of the repository/repositories and accession number(s) can be found below: NCBI [accession: PRJNA735102].

## Author Contributions

XG contributed to the investigation, software, data curation, formal analysis, and writing the original draft. PZ contributed to the investigation, methodology, isualization, and alidation. XZ contributed to the investigation, methodology, revision, and alidation. XC contributed to the conceptualization, funding acquisition, project administration, resources, and alidation. QZ contributed to the conceptualization, data curation, project administration, supervision, and validation. All authors contributed to the article and approved the submitted version.

## Conflict of Interest

The authors declare that the research was conducted in the absence of any commercial or financial relationships that could be construed as a potential conflict of interest.

## Footnotes

1 https://qiime.org

## References

[B1] Adegbeye M. J. Elghandour M. M. M. Y. Monroy J. C. Abegunde T. O. Salem A. Z. M. Barbabosa-Pliego A. (2019). Potential influence of Yucca extract as feed additive on greenhouse gases emission for a cleaner livestock and aquaculture farming-A review. *J. Clean. Prod.* 239 118074. 10.1016/j.jclepro.2019.118074

[B2] Andersen A. Z. Lauritsen F. R. Olsen L. F. (2005). On-line monitoring of CO_2_ production in *Lactococcus lactis* during physiological pH decrease using membrane inlet mass spectrometry with dynamic pH calibration. *Biotechnol. Bioeng.* 92 740–747. 10.1002/bit.20641 16224787

[B3] Araújo J. A. S. Almeida J. C. C. Reis R. A. Carvalho C. A. B. Barbero R. P. (2020). Harvest period and baking industry residue inclusion on production efficiency and chemical composition of tropical grass silage. *J. Clean. Prod.* 266 121953. 10.1016/j.jclepro.2020.121953

[B4] Bai J. Xie D. Wang M. Li Z. Guo X. (2020). Effects of antibacterial peptide-producing *Bacillus subtilis* and *Lactobacillus buchneri* on fermentation, aerobic stability, and microbial community of alfalfa silage. *Bioresource. Technol.* 315 123881. 10.1016/j.biortech.2020.123881 32731157

[B5] Cai Y. Ohmomo S. Ogawa M. Kumai S. (1997). Effect of NaCl-tolerant lactic acid bacteria and NaCl on the fermentation characteristics and aerobic stability of silage. *J. Appl. Microbiol.* 83 307–313. 10.1046/j.1365-2672.1997.00229.x 9351210

[B6] Chen M. Wolin M. J. (1977). Influence of CH_4_ production by *Methanobacterium ruminantium* on the fermentation of glucose and lactate by *Selenomonas ruminantium*. *Appl. Environ. Microb.* 34 756–759. 10.1128/aem.34.6.756-759.1977 PMC242743 596874

[B7] Converti A. Perego P. (2002). Use of carbon and energy balances in the study of the anaerobic metabolism of *Enterobacter aerogenes* at variable starting glucose concentrations. *Appl. Microbiol. Biotechnol.* 59 303–309. 10.1007/s00253-002-1009-5 12111162

[B8] de Souza Araújo E. Pimenta A. S. Feijó F. M. C. Castro R. V. O. Fasciotti M. Monteiro T. V. C. (2018). Antibacterial and antifungal activities of pyroligneous acid from wood of *Eucalyptus urograndis* and *Mimosa tenuiflora*. *J. Appl. Microbiol.* 124 85–96. 10.1111/jam.13626 29095556

[B9] Dunière L. Sindou J. Chaucheyras-Durand F. Chevallier I. Thévenot-Sergentet D. (2013). Silage processing and strategies to prevent persistence of undesirable microorganisms. *Anim. Feed. Sci. Tech.* 182 1–15. 10.1016/j.anifeedsci.2013.04.006

[B10] Emerson E. L. Weimer P. J. (2017). Fermentation of model hemicelluloses by *Prevotella* strains and *Butyrivibrio fibrisolvens* in pure culture and in ruminal enrichment cultures. *Appl. Microbiol. Biotechnol.* 101 4269–4278. 10.1007/s00253-017-8150-7 28180916

[B11] He L. Wang C. Xing Y. Zhou W. Pian R. Yang F. (2019). Dynamics of proteolysis, protease activity and bacterial community of *Neolamarckia cadamba* leaves silage and the effects of formic acid and *Lactobacillus farciminis*. *Bioresource. Technol.* 294 122127. 10.1016/j.biortech.2019.122127 31525585

[B12] He L. Zhou W. Xing Y. Pian R. Chen X. Zhang Q. (2020). Improving the quality of rice straw silage with *Moringa oleifera* leaves and propionic acid: Fermentation, nutrition, aerobic stability and microbial communities. *Bioresource. Technol.* 299 122579. 10.1016/j.biortech.2019.122579 31855660

[B13] Jung K. H. (2007). Growth inhibition effect of pyroligneous acid on pathogenic fungus, *Alternaria mali*, the agent of Alternaria blotch of apple. *Biotechnol. Bioprocess. Eng.* 12 318–322. 10.1007/BF02931111

[B14] Lee C. R. Kim C. Song Y. E. Im H. Oh Y. Park S. (2018). Co-culture-based biological carbon monoxide conversion by *Citrobacter amalonaticus* Y19 and *Sporomusa ovata* via a reducing-equivalent transfer mediator. *Bioresource. Technol.* 259 128–135. 10.1016/j.biortech.2018.02.129 29549832

[B15] Li C. Y. Zhou Y. L. Ji J. Gu C. T. (2016). Reclassification of *Enterobacter oryziphilus* and *Enterobacter oryzendophyticus* as *Kosakonia oryziphila* comb. nov. and *Kosakonia oryzendophytica* comb. nov. *Int. J. Syst. Evol. Micr.* 66 2780–2783. 10.1099/ijsem.0.001054 27045188

[B16] Li R. Narita R. Nishimura H. Marumoto S. Yamamoto S. P. Ouda R. (2018). Antiviral activity of phenolic derivatives in pyroligneous acid from hardwood, softwood, and bamboo. *ACS. Sustain. Chem. Eng.* 6 119–126. 10.1021/acssuschemeng.7b01265

[B17] Liu X. Li J. Cui X. Ji D. Xu Y. Chen T. (2020). Exogenous bamboo pyroligneous acid improves antioxidant capacity and primes defense responses of harvested apple fruit. *LWT-Food. Sci. Technol.* 134 110191. 10.1016/j.lwt.2020.110191

[B18] Liu Z. Liu Z. Li J. Xie N. Qin W. Feng W. (2020). Analysis on the development status of Chinese silage feed industry. *Journal of Grassland and Forage Science* 251 70–75.

[B19] Lo R. Turner M. S. Weeks M. Bansal N. (2016). Culture-independent bacterial community profiling of carbon dioxide treated raw milk. *Int. J. Food Microbiol.* 233 81–89. 10.1016/j.ijfoodmicro.2016.06.015 27344229

[B20] McDonald P. Henderson A. R. Heron S. J. E. (1991). *The Biochemistry of Silage, second ed^∗^.* UK: Chalcombe Publications, 10.1017/S0014479700023115

[B21] McEniry J. Forristal P. D. O’Kiely P. (2011). Gas composition of baled grass silage as influenced by the amount, stretch, colour and type of plastic stretch-film used to wrap the bales, and by the frequency of bale handling. *Grass Forage Sci.* 66 277–289.

[B22] Ni K. Zhao J. Zhu B. Su R. Pan Y. Liu X. (2018). Assessing the fermentation quality and microbial community of the mixed silage of forage soybean with crop corn or sorghum. *Bioresource. Technol.* 265 563–567. 10.1016/j.biortech.2018.05.097 29861298

[B23] Okyay T. O. Nguyen H. N. Castro S. L. Rodrigues D. F. (2016). CO_2_ sequestration by ureolytic microbial consortia through microbially-induced calcite precipitation. *Sci. Total. Environ.* 572 671–680. 10.1016/j.scitotenv.2016.06.199 27524723

[B24] Okyay T. O. Rodrigues D. F. (2015). Biotic and abiotic effects on CO_2_ sequestration during microbially-induced calcium carbonate precipitation. *FEMS. Microbiol. Ecol.* 91 fiv017. 10.1093/femsec/fiv017 25764465

[B25] Pahlow G. Muck R. Driehuis F. Oude Elferink S. Spoelstra S. (2003). “Microbiology of ensiling,” in *Silage science and technology agronomy*, eds Buxton D. R. Muck R. Harrison J. H. (USA: American Society of Anesthesiologists), 31–93. 10.2134/agronmonogr42.c2

[B26] Ray N. E. Maguire T. J. Al-Haj A. N. Henning M. C. Fulweiler R. W. (2019). Low greenhouse gas emissions from oyster aquaculture. *Environ. Sci. Technol*. 53 9118–9127. 10.1021/acs.est.9b02965 31295406

[B27] Suresh G. Pakdel H. Rouissi T. Brar S. K. Fliss I. Roy C. (2019). *In vitro* evaluation of antimicrobial efficacy of pyroligneous acid from softwood mixture. *Biotechnology Research and Innovation* 3 47–53. 10.1016/j.biori.2019.02.004

[B28] von Borzyskowski L. S. Carrillo M. Leupold S. Glatter T. Kiefer P. Weishaupt R. (2018). An engineered Calvin-Benson-Bassham cycle for carbon dioxide fixation in *Methylobacterium extorquens* AM1. *Metab. Eng.* 47 423–433. 10.1016/j.ymben.2018.04.003 29625224

[B29] Wang C. He L. Xing Y. Zhou W. Yang F. Chen X. (2019). Fermentation quality and microbial community of alfalfa and stylo silage mixed with *Moringa oleifera* leaves. *Bioresource. Technol.* 284 240–247. 10.1016/j.biortech.2019.03.129 30947138

[B30] Wang Y. He L. Xing Y. Zheng Y. Zhou W. Pian R. (2019). Dynamics of bacterial community and fermentation quality during ensiling of wilted and unwilted *Moringa oleifera* leaf silage with or without lactic acid bacterial inoculants. *mSphere*^∗^ 4 e341–e319. 10.1128/mSphere.00341-19 31391277 PMC6686226

[B31] Wang M. Lan X. Xu X. Fang Y. Singh B. P. Sardans J. (2020). Steel slag and biochar amendments decreased CO_2_ emissions by altering soil chemical properties and bacterial community structure over two-year in a subtropical paddy field. *Sci. Total. Environ.* 740 140403. 10.1016/j.scitotenv.2020.140403 32927559

[B32] Wang Y. Wang C. Zhou W. Yang F. Chen X. Zhang Q. (2018). Effects of wilting and *Lactobacillus plantarum* addition on the fermentation quality and microbial community of *Moringa oleifera* leaf silage. *Front. Microbiol.* 9:1817. 10.3389/fmicb.2018.01817 30127780 PMC6087751

[B33] Weinberg Z. G. Muck R. E. (1996). New trends and opportunities in the development and use of inoculants for silage. *FEMS. Microbiol. Rev.* 19 53–68.

[B34] Wu Z. Luo Y. Bao J. Luo Y. Yu Z. (2020). Additives affect the distribution of metabolic profile, microbial communities and antibiotic resistance genes in high-moisture sweet corn kernel silage. *Bioresource. Technol.* 315 123821. 10.1016/j.biortech.2020.123821 32683292

[B35] Zhai Y. Pérez-Díaz I. M. (2020). Contribution of *Leuconostocaceae* to CO_2_-mediated bloater defect in cucumber fermentation. *Food. Microbiol.* 91 103536. 10.1016/j.fm.2020.103536 32539962

[B36] Zhang L. Liu Y. Zhao R. Zhang C. Jiang W. Gu Y. (2020). Interactive regulation of formate dehydrogenase during CO_2_ fixation in gas-fermenting bacteria. *mBio* 11 e650–e620. 10.1128/mBio.00650-20 32817100 PMC7439476

[B37] Zhang Y. Wang X. Liu B. Liu Q. Zheng H. You X. (2020). Comparative study of individual and Co-Application of biochar and wood vinegar on blueberry fruit yield and nutritional quality. *Chemosphere* 246 125699. 10.1016/j.chemosphere.2019.125699 31884234

[B38] Zhang Q. Yu Z. Na R. S. (2018). Effects of different additives on fermentation quality and aerobic stability of *Leymus chinensis* silage. *Grass and Forage Science^∗^* 73 10.1111/gfs.12301

[B39] Zheng H. Wang R. Zhang Q. Zhao J. Li F. Luo X. (2020). Pyroligneous acid mitigated dissemination of antibiotic resistance genes in soil. *Environ. Int.* 145 106158. 10.1016/j.envint.2020.106158 33038622

